# Partner support and women's contraceptive use: insight from urban poor communities in Accra, Ghana

**DOI:** 10.1186/s12905-022-01799-7

**Published:** 2022-06-25

**Authors:** Martin Wiredu Agyekum, Elizabeth G. Henry, Mawuli Komla Kushitor, Akua Danquah Obeng-Dwamena, Caesar Agula, Patrick Opoku Asuming, Theophilus Toprah, Charles Agyei-Asabere, Iqbal Shah, Ayaga A. Bawah

**Affiliations:** 1 University of Ghana, Accra, Ghana; 2 University of Education, Winneba, Ghana; 3 Harvard TH Chan School of Public Health, Boston Massachusetts, USA; 4 University of Health and Allied Sciences, Ho, Ghana

**Keywords:** Partner, Contraceptive use, Modern methods, Traditional methods

## Abstract

**Background:**

Despite the benefits associated with contraceptive use, there is a low prevalence of contraceptive use in sub-Saharan Africa and Ghana. Previous studies have partly and consistently attributed the low prevalence of contraceptive use to partner opposition. However, little is known about the influence of men in contraceptive related choices of their partners, particularly within the context of urban poverty. This study examines the influences of partners on women’s contraceptive choices.

**Methods:**

The study utilized a cross-sectional household survey data of 1578 currently married women and women in a union of reproductive ages 16–44 years. Women who were pregnant and those trying to be pregnant were excluded from the analysis. The dependent variables for the study were current use of any contraceptive method, types of contraceptive methods and types of modern contraceptive methods. The independent variable for the study was a woman’s report of partner support in contraceptive related choices. A binary logistic regression model was used to examine the associations between partner support in contraceptive related choices and contraceptive use of women.

**Results:**

The results of the study show that partner support of contraceptive related choices has a significant influence on contraceptive use of women. Women who indicated support from their partners were more likely to be current users of any contraceptive method, yet were less likely to use modern contraceptive methods.

**Conclusion:**

The study highlights the need to involve men in family planning programs and research, as well as educating them on the various contraceptive modern methods and the side effects.

## Background

Globally, contraceptives remain an important public health intervention for preventing unintended pregnancies and related maternal mortality and morbidity [1–4]. Despite these benefits, studies show low use of contraceptives across sub-Saharan Africa (SSA) including Ghana, where the modern contraceptive prevalence rate is about 25% [5], a figure slightly lower than the regional (SSA) average. Modern contraceptive prevalence in SSA among married women or women in a union was estimated at 23.9% in 2012 and 28.5% in 2017 [6]. A variety of contextual factors such as service-related conditions, accessibility, availability and individual factors such as age, marital status, education, partner’s disapproval challenges the availability and use of contraceptives [7–9]. Even though there is almost universal awareness and actual availability of modern contraceptive methods, many women in the union do not use contraceptives due to their partners' objection and other personal reasons [10, 11]. Studies show that the low rate of contraceptive use in sub-Saharan Africa may be the result of partner disapproval of contraceptive use [12–14]. Limited choice of male contraceptives (such as condoms), perceived harmful side effects of female contraceptives and suspicions of spousal infidelity have also been identified as reasons for men’s objection to family planning methods [15–19]. The limited emphasis on men perhaps explains the scarcity of knowledge regarding the influence of men in the contraceptive decisions of their partners.

This study is informed by gender and power concepts which assert that power imbalances in sexual unions are explained by sexual division of labour, sexual division of power and cathexis. The interplay of these elements produces gender disparities and inequities that make women more vulnerable to their partners in their sexual and reproductive health [20]. Untill now, men retain and control the factors of production in many African contexts, women may therefore be subjugated to men, reducing their ability to assert themselves in sexual unions. In spite of these widely acknowledged power imbalances in sexual unions, family planning programs have mostly neglected the influence of men and how they specifically shape the use, non-use and type of family planning discussions in sexual unions [13]. Rather, family planning programs and research have focused mainly on women as the primary beneficiaries with less attention on men [21, 22]. There is evidence to show that men specifically have and do use their influence on contraceptive use in sexual unions [21, 22]. This influence is partly strengthened by ideologies of masculinity, gender roles and beliefs existing in both patrilineal and matrilineal societies which allow men control over women’s sexual and reproductive decisions and health [23, 24]. Studies on covert use of contraceptives are a sobering reminder of the power dynamics in sexual unions [1].

Male support for contraception may have an influence on women’s contraceptive use, types of contraceptive methods and types of modern contraceptive methods used. Evidence shows that partner support is associated with women’s contraceptive use [10, 11, 25]. However, results on the influence of partner support on the type of partner’s contraceptive method use are mixed. Whiles some studies have reported that partner support influences the use of modern contraceptive methods [8, 26], other studies have reported the influence of men on traditional methods due to limited knowledge on modern contraceptive methods, cost, accessibility and side effects of modern contraceptives methods [18, 19, 27]. Also, some women conceal contraceptive use despite their partners' disapproval to avoid tensions within relationships [15, 28, 29]. Balogun et al., [15] argued that there is an increase in modern contraceptive use when partners approve of contraceptive use for women.

Though there are many studies on contraceptive use in SSA and Ghana [12, 30–32], few studies in the sub-region and Ghana have examined partner support and contraceptive use among urban poor women [10, 33, 34]. Contextually, the concomitant rise in Africa’s urbanization and HIV/AIDS clarified the relationship between place and sexual activity on a broad population level. Deviating from the traditional development trajectories, Africa’s fast urbanization was not associated with economic development [35, 36]. For Accra, like most capital cities on the African continent, rapid population growth in urban centers was rather associated with deepening urban poverty. For example, while the levels of poverty had significantly reduced around the country in Ghana, urban poverty had intensified and widened. Research evidence from  five African cities including Accra, shows how urban poverty elevated risky sexual behaviours [35, 36]; particularly, lower age at first sex, low use of modern contraception and multiple sexual partners. Loose sexual unions, typified by short term co-habitation, another characteristic feature of urban poverty, amplify the risky sexual behaviours within these areas [35, 36].

In Ghana, studies on partner influence and contraceptive use especially among urban poor women are scarce [12, 37]. There is no study comprehensively examining partner influence on current contraceptive use, types of contraceptive methods, and types of modern contraceptive methods among urban poor women. Urban poor communities are characterized by high poverty levels, poor infrastructure and inadequate access to water and sanitation. Mostly, urban poor families are large and this may affect the utilization of health care services including limited access to family planning options [38]. In addition, the  disproportionately higher rates of unintended pregnancies in urban poor communities indicate that there is a high unmet need for family planning services [35]. While there is overwhelming evidence to show how urban poverty influences sexuality in an urban poor context, little is known about how partner support improves women’s use of modern contraceptives.

Understanding the influence of men on women’s contraceptive use could help increase the uptake of contraception of women in Ghana and prevent unwanted pregnancies among urban poor women. Also, the inclusion of males in family planning programmes and policy could help address misconceptions and myths about partners’ attitudes toward contraception. This study fills the gap in the literature by examining the influence of partner support in contraceptive use among urban poor women.

## Methods

### Study design and setting

The study utilized cross-sectional household survey data which was conducted between January and July 2018. This was a collaboration between Harvard T.H Chan School of Public Health and the Regional Institute for Population Studies, University of Ghana. The survey collected reproductive health information on women aged 16–44 years residing in two study sites within urban poor communities in Accra. The first study site included the coastal communities of Osu, Labadi, Teshie and Nungua, and the second included inland areas of Madina, Ashongman, and Agboba. The main economic activity for the communities along the coast is fishing, whiles for the community inland it is sales and services. The communities are characterized by low socioeconomic status and limited access to essential health services [39, 40]

### Sampling procedure and data collection

A multi-stage sampling method was used to create a representative sample of women in each of the two study sites. The first stage involved the selection of clusters from equally-sized sub-divisions of census enumeration areas derived from the Ghana Statistical Service’s census in the communities consisting of approximately 60 households [41]. This was followed by a complete listing of all households in the selected clusters that included at least one eligible woman. The second stage involved the random selection of approximately 25 households per cluster in which at least one eligible woman lived. The third and final stage involved random selection of one woman within the household for an interview if more than one eligible woman resided there. All women selected for the interview were introduced to the study by trained enumerators and their consent was sought. Participants who voluntarily provided written consent were enrolled. In all, 5,836 households were initially sampled, with 4,952 women eligible for the study, and 4,184 consented to the study. The questionnaire for the data collection consisted of individual and household characteristics, individual reproduction, family planning, pregnancy, marriage, fertility preference and contraception, and other health-related issues.

### Inclusion and exclusion criteria

The inclusion criteria were women currently married and those in union aged 16–44 years, who lived in the study area at the time of the interview and could communicate in English or local languages (Twi and Ga). For this study, we further restricted the analysis to women who had ever used any contraceptive method and were currently married or in a union producing a final analytic sample of 1578 women. Women who were pregnant and those trying to be pregnant were excluded from the analysis. They were excluded based on a series of questions. During the survey, the women were asked “*are you pregnant now?*” and those who were not pregnant were asked “*do you want to get pregnant now?*”. Based on the responses, we were able to identify those who were pregnant and those  trying to get pregnant.

### Ethical clearance

Ethical clearance for the study was obtained from the Ethics Review Committee of the Ghana Health Service (GHS-ERC #005/08/2017), the University of Ghana Ethics Committee for the Humanities (ERC #020/17–18) and the Harvard T.H Chan School of Public Health Institutional Review Board. Informed consent was obtained from each participant before they were interviewed. All participants provided written informed consent to participate in the study. The methods were carried out according to the guidelines specified and approved by the ethics committee. The purpose of the study including the general objectives, benefits, and risks of taking part in the study were explained to the participants. The participants were informed about the strict confidentiality and anonymity of their responses. They were also informed about their right to stop participating in the study at any point if they desired to do so. Those who agreed to be part of the study signed an informed consent form and, if they could not read or write, they provided a thumbprint to participate in the study.

## Measurement of variables

### Dependent variables

The dependent variables for the study include 1) current use of any contraceptive method, 2) type of current contraceptive method used, and 3) type of current modern contraceptive method. Current use of any contraceptive method was defined as the use of any current family planning method, measured as a dichotomous variable “current users” versus “non-current user”. Type of current contraceptive method was classified as a traditional method versus a modern method. Women who used withdrawal, calendar and rhythm methods were grouped as “traditional method” while those who used IUD, implants, injectable, oral contraceptive pills, emergency contraceptives, condoms and Lactational Amenorrhea Method (LAM) were classified as “modern contraceptive method users” [37]. The type of modern contraceptive method used was categorized into “long-acting reversible contraception” (LARC) and “short term” methods. The LARC methods comprised of IUD and implants. The short-term methods included oral contraceptive pills, condoms, emergency contraceptives, injectables and LAM.

### Independent variable

The independent variable for the study was a woman’s report of partner support in contraceptive related choices. During the survey, all women married or in union were asked “Does your husband/partner support you in your choices related to contraceptive use?” with the response options “Yes” and “No”. Women who had support from their partners were classified as “Yes” and those with no support from their partners were classified as “No”.

### Control variables

In addition, other sociodemographic variables related to the outcome variables were included and controlled for. These variables were included based on the literature review [8, 10, 12, 19]. The variables include age, educational level, religion, employment status, household wealth quintile, ethnicity, health decision-making, children ever born, partner illiteracy and family planning counselling. Age of women was coded as 16–19, 20–24, 25–29, 30–34, 35–39 and 40–44. The educational level of women was classified as level attained: no education, primary, junior high school, secondary and higher education. Religion was categorized as Pentecostal/charismatic, Protestant, Orthodox, other Christians, Moslem and other religious affiliation. Employment status was classified as employed regardless of the sector either formal or informal, and unemployed. Also, the household wealth quintile was classified as poorest, poorer, middle, richer and richest relative to the Ghana Demographic and Health Survey (GDHS) urban household wealth quintiles. Ethnicity was categorized as Akan, Ga/Dangme, Ewe, Mole-Dagbani and other ethnic groups. Health decision-making was grouped as respondents only, husband/partner only and husband/partner jointly. Also, children ever born was measured as a continuous variable. Receiving family planning counselling within the last two years at the health facility was classified as yes and no. Partner literacy was coded as yes and no in response to the question “Can your (husband/partner) read a phrase/sentence in English?”.

### Method of analysis

Stata version 15 was used for the analysis. The data were analyzed at both the univariate and multivariate levels. Descriptive statistics such as frequency and percentage,  and mean and standard deviation were used to describe the proportion of each variable for categorical and continuous variables, respectively. Binary logistic regression was used to examine the associations between partner support in contraceptive-related choices and contraceptive use reported by women. Three separate models were fit to examine the associations between partner support and contraceptive behavior of women. Adjusted odds ratios (AORs) and 95% confidence intervals were also used to describe and interpret the findings of the study. A p-value less than 0.05 was used for statistical significance and weighting was applied.

## Results

### Description of background characteristics

Table 1 shows the background characteristics of women. From Table 1, about 82.9.% women indicated that their partners supported their choices of contraceptive use while 17.1% reported that their partners did not support them in their contraceptive choices. Just over a quarter of the respondents (27.4%) were aged 30–34, 22.0% were aged 25–29, and the smallest proportion (1.0%) were aged 16–19. With regards to education, the majority (77.4%) of the women had at least junior high school education. Specifically, a higher proportion (44.1%) of the women had junior high school education and about 12.5% had tertiary education. Slightly more than half (53.9%) of the women were affiliated to the Pentecostal/charismatic religious denominations, and the least proportion 2.2% belong to other religious faiths. About one third (35.7%) of the women belong to the Akan ethnic group. In addition, only 26.4% belonged to the two poorest household wealth quintiles. With regards to employment status, about eight out of ten (88.1%) women reported being currently employed. Also, slightly more than half (50.4%) of the women jointly took decisions with their partners regarding their health issues. In addition, nearly all women reported that their partners/husbands were literate.

**Table 1:** Percentage distribution of socio-demographic characteristics of respondents

Variables	Frequency	Percentage	Variables	Frequency	Percentage
*Partner support in contraceptives*			*Household wealth quintile of woman*		
No	270	17.1	Poorest	104	6.6
Yes	1308	82.9	Poorer	313	19.8
			Middle	569	36.1
*Age of woman*			Richer	462	29.3
16–19	15	1.0	Richest	130	8.2
20–24	165	10.5	*Ethnicity of woman*		
25–29	347	22.0	Akan	563	35.7
30–34	432	27.4	Ga/Adangbe	501	31.7
35–39	357	22.6	Ewe	282	17.9
40–44	262	16.5	Mole-Dagbani	92	5.8
*Educational level attained by a woman*			Other Ethnic groups	140	8.9
No education	105	6.7	*Health decision-making*		
Primary	251	15.9	Respondents only	605	38.3
Junior High	696	44.1	Husband/partner only	178	11.3
Secondary	329	20.8	Husband/partner jointly	795	50.4
Higher	197	12.5	*Received any family planning counselling at a health facility in the past two years*		
*Religion of woman*			Yes	585	37.1
Pentecostal/Charismatic	850	53.9	No	993	62.9
Protestants	172	10.9	*Partner literacy*		
Orthodox	85	5.3	Yes	1480	93.8
Other Christians	279	17.7	No	98	6.2
Moslem	157	10.0	*Total*	*1578*	*100.0*
Other	35	2.2			
*Employment status of women*			*Children Ever Born (Parity)*	Min (max)	Mean (std dev)
Unemployed	188	11.9		0 (11)	2.37 (1.44)
Employed	1390	88.1			
Total	1578	100.0			

### Description of dependent variables

Table 2 shows the description of the dependent variables. Slightly more than half (53.5%) of women currently reported using a method to prevent pregnancy. About 58.1% of the women using any method were currently using modern contraceptive methods. However, the prevalence of modern contraceptive use among the general population was 25.8%. From the sub-population of women using any modern contraceptive method to prevent pregnancy, the majority (63.7%) of them were using short term methods.

**Table 2:** Percentage distribution of contraceptive uptake among urban poor women

Dependent variables	Frequency	Percentage	Dependent variables	Frequency	Percentage
*Current users of contraceptive methods*			*Types of modern contraceptive methods use*		
No	734	46.5	Short term methods	312	63.7
Yes	844	53.5	long term methods	178	36.3
Total	1578	100	Total	490	100
*Types of contraceptive methods use (among users)*					
Traditional	354	41.9			
Modern method	490	58.1			
Total	844	100			

### Method types and partner support in contraceptive related choices

Table 3 shows the method types used by women depending on their partner’s support in contraceptive-related choices. The results show that the highest proportion (31.2%) of partners had no support for implants and the least proportion (1.3%) had no support for emergency contraceptives. On the other hand, most of the partners (18.6%) supported the use of the rhythm method while the least proportion of the partners (2.5%) support emergency contraceptives.

**Table 3:** Percentage distribution of method types and partner support in contraceptive related choices

Variables	Partner support in contraceptive related choices		
Method types	No	Yes	Total
*Long term methods*			
IUD	2 (2.6)	23 (3.0)	25 (3.0)
Implants	24 (31.2)	129 (16.8)	153 (18.1)
*Short term methods*			
Pills	6 (7.8)	58 (7.6)	64 (7.6)
Condom	3 (3.9)	51 (6.6)	54 (6.4)
Emergency contraceptive	1 (1.3)	19 (2.5)	20 (2.4)
Injectable	17 (22.1)	125 (16.3)	142 (16.8)
Lactational Amenorrhea Method (LAM)	4 (5.2)	28 (3.7)	32 (3.8)
*Traditional methods*			
Standard days/Calendar Method	5 (6.4)	71 (9.3)	76 (9.0)
Rhythm	10 (13.0)	143 (18.6)	153 (18.1)
Withdrawal	5 (6.5)	120 (15.6)	125 (14.8)
Total	77	767	844

Table 4 illustrates factors associated with contraceptive uptake among urban poor women who were married/in-union in Accra, Ghana. The multivariate binary regression (model 1), which adjusts for all other variables, shows that partner support in contraceptive choices was significantly associated with current contraceptive use and modern method contraceptive use. Women who had support from their partners were more likely (AOR = 4.36; CI 95%: 3.05–6.25) to currently use any contraceptive method than those with reportedly no support from their partners/husbands. The odds (AOR = 0.39; CI 95%: 0.19–0.79) of currently using a modern contraceptive method was lower for women who received support from their partners than those with no support in Model 2.

**Table 4:** Factors associated with contraceptive use among urban poor women

	Factors associated with current use of contraceptive methods (*N* = 1578) Model 1		Factors associated with current modern contraceptive methods (*N* = 844) Model 2		Factors associated with current modern long-acting reversible methods (*N* = 490) Model 3	
	Adjusted Odds Ratio (95% CI)	*P*-value	Adjusted Odds Ratio (95% CI)	*P*-value	Adjusted Odds Ratio (95% CI)	*P*-value
*Partner support of contraceptive related choices*						
No (REF)						
Yes	4.36 (3.05–6.25)	**0.00**	0.39 (0.19–0.79)	**0.01**	0.67 (0.33–1.34)	0.25
*Age of women*						
16–19 (REF)						
20–24	1.22 (0.35–4.28)	0.75	1.72 (0.39–7.65)	0.47	1.15 (0.16–8.56)	0.89
25–29	0.77 (0.24–2.55)	0.67	1.54 (0.37–6.47)	0.55	0.39 (0.05–3.13)	0.37
30–34	0.55 (0.15–1.97)	0.36	0.88 (0.21–3.67)	0.86	0.25 (0.03–2.11)	0.20
35–39	0.48 (0.13–1.75)	0.27	0.74 (0.16–3.47)	0.70	0.40 (0.04–3.62)	0.42
40–44	0.21 (0.06–0.77)	**0.02**	0.54 (0.11–2.60)	0.44	0.28 (0.03–2.38)	0.24
*Educational level of women*						
No education (REF)						
Primary	1.06 (0.52–2.16)	0.88	0.68 (0.27–1.70)	0.40	0.65 (0.20–2.16)	0.48
Middle	1.45 (0.80–2.62)	0.22	0.94 (0.28–2.33)	0.90	0.58 (0.18–1.87)	0.36
Secondary	1.18 (0.60–2.35)	0.63	1.11 (0.41–3.03)	0.84	1.10 (0.32–3.76)	0.89
Higher	2.02 (0.94–4.37)	0.07	0.75 (0.26–2.10)	0.58	0.89 (0.21–3.83)	0.88
*Religion of women*						
Pentecostal/Charismatic (REF)						
Protestants	0.89 (0.54–1.46)	0.65	1.11(0.62–1.99)	0.72	0.80 (0.32–2.00)	0.63
Orthodox	0.92 (0.50–1.67)	0.78	1.27 (0.56–2.90)	0.56	0.42 (0.15–1.13)	0.09
Other Christians	1.04 (0.72–1.50)	0.84	0.70 (0.41–1.19)	0.18	0.59 (0.29–1.22)	0.15
Moslem	0.50 (0.25–1.00)	0.05	0.47 (0.20–1.10)	0.08		
Other	1.39 (0.48–4.00)	0.54	1.13 (0.39–3.32)	0.82	1.74 (0.73–4.16)	0.21
*Ethnicity of women*						
Akan (RF)						
Ga/Adangme	0.88 (0.64–1.20)	0.41	1.50(0.94–2.39)	0.09	2.33 (1.24–4.37)	**0.01**
Ewe	1.51 (1.06–2.15)	**0.02**	1.26 (0.78–2.05)	0.34	2.78 (1.40–5.50)	**0.00**
Mole-Dagbani	0.92 (0.39–2.18)	0.84	3.53 (1.27–9.80)	**0.02**	2.37 (0.78–7.20)	0.13
Other Ethnic groups	0.87 (0.48–1.58)	0.65	2.96 (1.22–7.20)	**0.02**	1.90 (0.64–5.70)	0.25
*Household wealth quintile*						
Poorest (RF)						
Poorer	1.22 (0.73–2.04)	0.45	0.97 (0.46–2.08)	0.94	0.73 (0.29–1.86)	0.51
Middle	1.69 (1.07–2.66)	**0.02**	1.52 (0.80–2.91)	0.20	0.43 (0.18–1.06)	0.07
Richer	1.65 (1.00–2.70)	0.05	1.20 (0.57–2.52)	0.63	0.84 (0.33–2.13)	0.71
Richest	1.48 (0.76–2.88)	0.25	0.62 (0.24–1.57)	0.31	0.32 (0.07–1.55)	0.16
Children ever born (Parity)	1.41 (1.27–1.58)	**0.00**	1.15 (0.96–1.38)	0.14	1.19 (0.96–1.49)	0.12
*Employment status of women*						
Unemployed (RF)						
Employed	0.91 (0.59–1.43)	0.69	1.35 (0.72–2.52)	0.35	1.93 (0.90–4.15)	0.09
*Health decision-making*						
Respondents only (RF)						
Husband/Partner only	0.65 (0.42–1.00)	0.05	1.86 (0.88–3.94)	0.11	0.62 (0.26–1.49)	0.28
Husband/partner jointly	0.56 (0.41–0.75)	**0.00**	3.05 (1.99–4.67)	**0.00**	1.05 (0.62–1.77)	0.85
*Family planning counselling of women*						
Received counselling (RF)						
No counselling	0.98 (0.75–1.28)	0.86	0.76 (0.52–1.11)	0.15	1.05 (0.65–1.69)	0.84
*Partner literacy*						
Yes (RF)						
No	1.39 (0.71–2.70)	0.34	2.43 (0.97–6.10)	0.06	1.21 (0.49–2.97)	0.68
_cons	0.22	0.04	0.92	0.94	0.93	0.96

Significant *p* values were boldened to make them stand out

In addition, when all other variables were controlled for in the model, other variables that were associated with the current use of contraceptives were the age of women, ethnicity of women, household wealth quintile, children ever born and health decision. Women who were 40–44 years were less likely (AOR = 0.21; CI 95%: 0.06–0.77) to currently use a contraceptive method compared to women aged 16–19 years. Women belonging to the Ewe ethnic group were more (AOR = 1.51; CI95%: 1.06–2.15) likely to be current users of contraceptives than women belonging to the Akan ethnic group. Also, women who belong to the middle household wealth quintile were more likely (AOR = 1.69; CI 95%: 1.07–2.66) to be current users of any contraceptive method than those who belong to the poorest household wealth quintile. Also, as the number of children by women increased, the odds (AOR = 1.44; CI 95%: 1.27–1.58) of women being a current user of any contraceptive method increased. For decision-making, women who take decisions on health with their partners were less likely (AOR = 0.56; CI 95%: 0.41–0.75) to be current users of any contraceptive method relative to those who make decisions alone.

Furthermore, in Model 2, ethnicity of women and decisions on health were associated with the current modern method of contraceptive use. Mole-Dagbani women were more likely (AOR = 3.53; CI 95%: 1.27–9.80) to currently use modern contraceptive methods compared to women belonging to the Akan ethnic group. In addition, women belonging to other ethnic groups were more likely (AOR = 2.96; CI 95%: 1.22–7.20) to currently use modern contraceptive methods compared to women belonging to the Akan ethnic group. Women who jointly decide on their health with their partners were more likely (AOR = 3.05; CI 95% 1.99–4.67) to currently use modern contraceptive methods compared to women who take health-related decisions alone.

Moreover, only ethnicity of women was associated with currently using a modern long-acting reversible contraceptive method, after controlling for other variables in model 3. Women belonging to Ga/Adangme ethnic group were more likely (AOR = 2.33; CI 95%: 1.24–4.37) to currently use modern long-acting reversible contraceptive methods compared to women belonging to the Akan ethnic group. In addition, women belonging to the Ewe ethnic group were more likely (AOR = 2.78 CI 95%: 1.40–5.30) to currently use modern long-acting reversible contraceptive methods compared to women belonging to the Akan ethnic group.

## Discussion

Drawing on cross-sectional data among urban poor women in Ghana, this study examined the influence of husbands'/men’s support on their partners’ contraceptive use at different levels. This study supports other evidence that shows the importance of men in women’s contraceptive use and the need to include men in family planning programs. Previous studies have examined the influence of men's/husbands' support on contraceptive use and concluded that married women or women in union mostly require the support of their partners in contraceptive use [10, 13, 15]. Most of these studies were not done in urban poor communities and do not provide insight on the influence of the complexity of contraceptive use by women at different levels. This study contributes to knowledge by providing an understanding of the influence of husbands/men on the almost stepwise progress of contraceptive use at different levels (current contraceptive use, type of contraceptive use and type of modern contraceptive use) within urban poor communities in Accra. The results of this study highlight two main points for consideration by policymakers and researchers who are looking to address the unmet need for contraception and improve contraceptive uptake among married couples or women in union in the urban areas of Ghana and similar contexts. First, in urban poor communities in Ghana, most women perceive that their partners support them in contraceptive use and this, in turn, is associated with not only women’s actual use of contraceptives but also with the type of contraceptives they use. Secondly, the support of husbands/men in contraceptive-related choices reduces the likelihood of women using modern contraceptive methods.

The findings of this study showed that husband/partner support in contraceptive-related choices are associated with the current use of any contraceptive method by women. This is similar to findings of other studies [10, 13, 15] which have reported partner/husband support for contraceptive use. The results imply that women’s decision to use contraceptives was influenced by their partners' involvement in decision-making. The findings of the study may be supported by cultural and gendered norms where men have more decision-making power compared to women in reproductive issues. This is most entrenched in urban poor communities where many women have low incomes, low education and where there is no family support system to take decisions on their own which makes men the decision-makers on reproductive health. The findings of this study are also supported by the gender and power theory which argued that men have control over their partners’ sexual and reproductive health. The control therefore determines the use of any contraceptive method by their partners. Also, the power imbalance including the patriarchal system creates dominancy of  men which enables them to exert control over their partners' contraceptive choice. Most of the study communities are characterized by dominant masculinity norms which gives more power to men in reproductive health decisions including contraceptive use. Although some notions of femininity view pregnancy prevention within the domain of women, Ganle et al. [24], argued that gender-based power imbalance and gender inequality give more power to men which limits women’s autonomy. Women sometimes require permission from their husbands to utilize health care services in various parts of Ghana. As a result, they may resort to covert use without their partners' support. This, therefore, underscores the power imbalance in unions.

Also, the results demonstrate that men have a preference for traditional methods of contraception compared to modern methods. Partners may prefer traditional contraceptive methods such as withdrawal and rhythm methods because it requires the joint participation of the couple in its use and is devoid of the side effects of modern methods reported by some women [18]. Furthermore, from the men’s perspective, the use of traditional contraceptive methods could deter women from engaging in extramarital affairs mostly in urban poor settings for economic reasons. In addition, men’s limited knowledge of modern contraceptives could explain their preference for traditional contraceptive methods. This limited knowledge among urban poor people could account for false beliefs and men’s fear of harmful side effects of modern contraceptives. This is because men have limited access to contraceptive knowledge compared to women who are frequently exposed to the hospital environment during pre—and post-natal periods of pregnancy and childbirth [18, 42, 43]. In addition, the cost of modern contraceptives could also account for the husband’s preference for traditional methods, and the deterrence of both the men  and women  from using modern contraceptives [15, 44]. Couples in urban poor communities who are mostly engaged in low-income jobs may not be able to afford modern contraceptives such as IUD, implants and injectables due to their low socioeconomic background and may be forced to use traditional methods such as withdrawal and rhythm methods which are free. These factors, therefore, do not provide a comprehensive understanding to inform husbands on modern contraceptives thereby limiting the use of modern contraceptives.

The findings of the study show that there was no significant difference between partner support in contraceptive-related choices and the type of modern contraceptive method used. The statistically non-significant results could be a result of the husband’s preference for traditional methods or perhaps other factors that we did not examine in our study that is related to the decision-making around the type of modern method.

The results from this study illustrate perhaps a more nuanced understanding of how male support for contraceptive choices may influence their partner’s overall use, but also determine the effectiveness of the method chosen. The findings of this study are supported by the Gender and Power theory which explains that sexual division of labour, sexual division of power and the structure of cathexis produce greater inequities between men and women. These inequities make men exert a greater influence on their partners' sexual and reproductive health. In this study, most men exert greater support on their partners’ contraceptive-related choices and their influence determine the type of contraceptive that their partners use. The power imbalances and the patriarchal system sometimes make it difficult for women to bargain or have dominion in their sexual and reproductive health, thereby paving the way for men to exert an influence on their sexual and reproductive health.

This study is very relevant to the inclusion of men in family planning programs as the result shows that the support of men leads to the use of less effective family planning methods (traditional methods) by their partners. Contraceptive related choices have consequences on increasing infertility as well as maternal morbidity and mortality due to unintended pregnancies. The study is a cross-sectional study, hence causality could not be established. Findings from this study could inform policymakers to include men in family planning programs. Provision of family planning education needs to be strategized and information on family planning in urban poor settings should be intensified as policy targets men on family planning counselling.

## Conclusion

The study contributes to understanding the influence of male partners in women’s contraceptive use. The results show that partner support influences women’s current contraceptive use but less use of modern methods, which are the most effective at preventing pregnancy. The study highlights the need to involve men in family planning programs and research as well as educating them on the need to use modern contraceptive methods as it is more effective than traditional methods. Couples should be counselled on the management of contraceptive side effects as it may help to allay fears of debilitating side effects of modern contraceptives, and to quell myths associated with modern contraceptive use. There should be an improvement in accessibility of contraceptives in urban poor communities as well as improving on family planning education and information to increase uptake of modern contraceptives.

## Data Availability

The dataset for this study is available from the corresponding author upon reasonable request.
